# Monitoring the Italian Home Palliative Care Services

**DOI:** 10.3390/healthcare7010004

**Published:** 2019-01-02

**Authors:** Gianlorenzo Scaccabarozzi, Pietro Giorgio Lovaglio, Fabrizio Limonta, Carlo Peruselli, Mariadonata Bellentani, Matteo Crippa

**Affiliations:** 1 Department of Frailty, Local social health authority (ASST) Lecco, Local Network of Palliative care, Largo Mandic 1, 23807 Merate, Italy; g.scaccabarozzi@asst-lecco.it; 2 Department of Statistics and Quantitative Methods, University Bicocca-Milan, Via Bicocca degli Arcimboldi 8, 20126 Milan, Italy; 3 Health Territorial Agency (ATS) Montagna, Via Nazario Sauro 38, 23100 Sondrio, Italy; f.limonta@ats-montagna.it; 4 Foundation Maruzza Lefebvre D’Ovidio, Via del Nuoto 11, 00135 Rome, Italy; carlo.peruselli@gmail.com; 5 General Direction of Health Planning of the Italian Ministry of Health, 00144 Rome, Italy; md.bellentani@sanita.it; 6 Fondazione Floriani Via Privata Nino Bonnet 2, 20154 Milan, Italy; m.crippa@fondazionefloriani.eu

**Keywords:** palliative care, best practices, Italian National Observatory, home palliative care units, quality of the care

## Abstract

Background: In Italy, there currently is a lack of reliable and consistent data on home palliative care provided to people near death. Objectives: Monitoring the activities of the Italian Home Palliative Care Services, according to the 2014 national data collection program entitled “Observatory of Best Practices in Palliative Care” and providing process/outcome measures on a subsample (Best Practice Panel), on regulatory standards and on complete/reliable activity data. Design: A data collection web portal using two voluntary internet-based questionnaires in order to retrospectively identify the main care activity data provided within the year 2013 by Home care units. In the Best Practice Panel and International best practices, eligibility and quality measures refer to the national standards of the NL 38/2010. Setting/Subject: Home Palliative Care Services (HPCSs) that provided care from January to December 2013. Results: 118 Home care units were monitored, globally accounting for 40,955 assisted patients within the year 2013 (38,384 cancer patients); 56 (47.5% of 118) were admitted in the Best Practice Panel. Non-cancer (5%) and pediatric (0.4%) patients represented negligible percentages of frail care patients, and a majority of patients died at home (respectively nearly 75% and 80% of cancer and non-cancer patients). Conclusion: The study demonstrated the feasibility of the collection of certified data from Home care services through a web-based system. Only 80% of the facilities met the requirements provided by the Italian NL 38/2010. Moreover, the extension of the palliative care services provided to frail non-cancer and pediatric patients, affected by complex and advanced chronic conditions, is still inadequate in Italy.

## 1. Introduction

Out of a population of approximately 60.5 million inhabitants, around 613,000 people die each year in Italy, with a lot of women living up to 84.5 years and men up to 79.5 years. In 2012, the five most common causes of death were ischemic heart disease (12%), cerebrovascular disease (10%), other heart disease (8%), cancer (6%) and hypertensive disease (5%) [1].

Using the methods provided in literature [2,3,4] and the official 2012 Italian data on the causes of death, we estimated that chronic diseases are responsible for 77% of all deaths: consistently with national data, it can be estimated that about 250,000–470,000 people are likely to need Palliative Care (PC) in Italy each year.

Palliative care incorporates both near death and hospice care (for patients who are terminally ill and with a life expectancy of less than 6 months), and broadens traditional disease models by focusing more on enhancing the quality of life, on optimizing functions and on fostering near death care through decision-making processes and by providing emotional and spiritual support to patients and their families [5].

Evidence shows that promoting the provision of at-home PC to people near death leads to better outcomes, wherein dying at home is recognized as most significant in terms of improving the quality of life, reducing symptoms or even prolonging survival, also with regard to the wide range of care options available to patients and to the most cost-effective use of resources. [6,7,8,9,10]

The Italian National Healthcare System is tax-funded, both by public and private providers, and mainly managed by 21 Regions and the autonomous provinces. Each Region then has its Local Health Agencies (ASLs), which are in charge of providing care in hospitals, at home and in residential structures for the elderly.

PC is provided by the National Healthcare System (NHS) and was officially recognized by the National Law 38/2010 [11]; the number of palliative care units has been in continuous expansion since the mid-2000s and provide advanced home care, inpatient care (e.g., hospice) and consulting services.

Until recently, palliative care concerned almost exclusively adult/cancer patients (in 2010, 4.4% of the Italian population diagnosed with cancer was considered a potential user of the palliative care services) in Italy, whereas patients with progressive end-stage disorders (such as heart failure, chronic obstructive pulmonary disease and dementia) were denied access to these services, despite their prognosis and symptoms were either comparable to or worse than those of many cancer patients.

The NL 38/2010, issued to guarantee the right of access to PC, was introduced to increase access to palliative care for all patients with severe disabilities (especially non-cancer and pediatric patients with advanced and complex health conditions) and to allow them to select the care and death setting.

To this end, the NL 38/2010 promotes continuity of care among the different care settings of the NHS (at home, in hospices, in hospitals or in residential facilities). 

Moreover, the law provides opinions and guidelines on the priority settings and on how to implement both the National Palliative Care Programs and National Cancer Control Programs, by addressing, among others, suitable policies, adequate drug supplies, the training of health professionals and the implementation of palliative care services, at all levels, in order to increase awareness on the quality of care provided. 

Different Institutional Organizations, Scientific Societies, Nations and Scientific Committees [5,11,12,13,14,15] have implemented norms and standards to ensure qualitative palliative care procedures and to provide quality indicators to monitor them.

Many recent initiatives have focused on the efforts that many countries have made to improve the access and costs of palliative care and to monitor progress, also by illustrating the challenges and drawbacks with regard to the policies and infrastructures. Relevant examples of this were presented by the European Association for Palliative Care [16,17], by the Worldwide Palliative Care Alliance, by the Word Health Organization [18], by the Economist [19] and in literature reviews [20,21,22,23]. 

However, these experiences mainly focus on the state-of-art of palliative care at an individual country level, while the reports on the Italian system often only refer to the regulatory aspects without considering the daily practices of the PC facilities. 

In Italy, the literature does not monitor the palliative care treatments administered by the health facilities to patients in term of access and patients’ preferences, essentially due to a lack of official data, especially in the outpatient and community settings. 

In this perspective, there is also an imperative need to assess and to continuously improve the quality of care of the Italian PC facilities in daily practice and, hence, to develop monitoring systems based on measurable outcome/process indicators in order to ensure the timely and accurate dissemination of the data required for the implementation of best practice programs [24,25,26].

In order to address these challenges, the Italian Ministry of Health has been promoting a comprehensive research program, entitled “Observatory of Best Practices in Palliative Care” (OBPPC), since 2012. Inspired by International PC best practices, the program aimed at providing quality, reliable and timely data on Home Palliative Care Services (HPCSs), e.g., the facilities administering PC in the home setting, to help them improve their practices and to define a common clinical language for streamlining their intercommunication. 

HPCSs, which must satisfy some regulatory standards provided by the NL 38/2010, are composed of multi-professional teams created to ensure medical, nursing, rehabilitation and psychological services, as well as social, protective and spiritual support in favor of people with chronic and progressive diseases in a defined local area and for whom the evidence-based treatments do not adequately, effectively and significantly stabilize the disease.

HPCSs may deliver the primary palliative care (GPs and PC community nursing teams) or specialized palliative care (PC multidisciplinary teams) services of both public and private organizations.

The OBPPC program started in 2012 as a scientific research project aimed to identify all HPCSs active since 2011 across the Italian territory. In particular, Agenas and the Ministry of health, identified health structures that used both the administrative registers of Local Health Agencies (for public facilities) and the institutional registers of scientific societies (Italian Society of palliative care and Italian society of General Medicine) and of honorable and representative non-profit associations (the Italian Federation for Palliative care and Floriani Foundation), for private facilities.

In 2014, the OBPPC mainly continued to operate as a data collection program using an institutional web portal. 

However, no effort has been made to document the daily practices of the HPCSs registered in the voluntary OBPPC portal with regard to the implementation of the national law and to the quality of care provided according to the best practice standards, such as access to care and patients’ preferences in the final stages of life.

To this end, at least two aspects of the data of the OBPPC portal must be evaluated in order to achieve a rigorous assessment of the palliative care practices in Italy: (1) the regulatory standards provided for the administration of at-home PC for the HPCSs registered in the OBPPC portal; (2) the reliability and quality of the data provided. 

The present paper, the first in Italy to report data on the activities and care provided by the HPCSs in 2013, has two main objectives: (1) to assess if the care practices of the HPCSs registered in the OBPPC comply with the recommendations of the NL 38/2010; and (2) to present key process/outcome measures on a selected panel of HPCSs, called the Best Practice Panel, that comply with the regulatory standards and that provided accurate and reliable data. 

## 2. Methods

### 2.1. Data Collection

The voluntary registration to the OBPPC exclusively concerned Italian HPCSs and did not include acute care (hospital palliative care) and long-term care services (Nursing homes, residential facilities, Hospices).

A Steering Committee (made up of PC physicians, epidemiologists, national and regional stakeholders, managers and scholars), coordinated by the National Agency for the Regional Health Services (Agenas), provided guidelines and methods for both the study and collection of data. 

In 2014, the OBPPC call collected data on a web platform by means of two questionnaires: the general information, structure/process and activity data of HPCSs related to the patients assisted at home each year, to the workload of physicians and nurses and to the amount of home visits. The web platform allowed the institutional managers of HPCSs (identified across the Italian territory and contacted by Agenas) to record their answers online.

All information, collected at the HPCS level (aggregated data), was officially certified and transmitted to Agenas. 

Standard indicators useful in identifying HPCSs according to the registered HPCSs were provided by the NL38/2010, whereas the key quality process/outcome measures used to monitor the quality were inspired by the recommendations of international PC Institutions, Committees and Scientific Societies and, especially, by the guidelines developed by the National Institute for Clinical Excellence in the UK [27]. 

### 2.2. Admission Requirements of the Best Practice Panel

Four stages must be completed in order to ensure the inclusion of registered HPCSs within the Best Practice Panel.

First, each HPCS must comply with some of the regulatory standards required by law 38/2010 (Regulatory requirements, Stage 1) in order to achieve the HPCS status; second, the HPCSs have to provide an entire set of activity data related to the yearly volumes (minimum dataset, Stage 2); the standards and indicators involved at these two stages are listed in the upper part of Table 1, which also contains the descriptive results of all registered HPCSs called in 2014. 

The last two stages of the Best Practice Panel admission process deal with the analysis of the quality of data.

Stage 3 focuses on the reliability of the data provided by the HPCSs (that made it through Stage 2), by analyzing six key indicators K1–K6 based on the minimum dataset provided; specifically, we check, for each key indicators, possible HPCS outliers, by investigating the position of a HPCS with respect to the distribution of all HPCSs. A Boxplot is used as a statistical benchmark to identify the “mild” and “extreme” outliers. Here, only the very extreme outlier cases were considered: extreme outliers refer to any score beyond the extreme interval (Q1 − 3 × IQ; Q3 + 3 × IQ), called “outer fence,” where IQ = (Q3 − Q1) is the interquartile range, e.g., the difference between the third (Q3) and the first (Q1) quartile of the distribution indicator [28].

The upper part of Table 2 shows the key indicators K1–K6 involved in Stage 3 and the formulas used to calculate them. With regard to the key indicators, K1–K4 (assisted cancer patients per physician/nurse and to home visits per deceased cancer patients) are calculated by normalizing the amount of the HPCSs’ workforces according to the full-time equivalent physicians and nurses employed weekly (using 38 and 36 h per week, respectively, and the 44.6 equivalent weeks in the year, based on the National Contract for health professionals), whereas for the calculation of K5 and K6, the mean length of stay (weeks) at the HPCS level was considered as a representative value for all patients managed by a HPCS. 

Moreover, Stage 4 adds further evidence on the quality of the data provided by the HPCS by identifying possible outliers in a bivariate relationship between two indicators, extending the un-bivariate outlier analysis presented in Stage 3. 

Specifically, we explored the robustness of the relationship between the amount of nurse and physician weekly visits recorded per deceased cancer patient and the case-mix of deceased cancer patients assisted by each HPCS over the year under study. 

To this end, in the Italian context, the Intensity Care Coefficient (that at patient level is equivalent to the number of days with at least one home visit divided by the duration of care, in days) is becoming mandatory both at the patient and HPCS level, since the effectiveness of the NL 38/2010, and is the best/available severity indicator (both at a clinical and social level) of the patient’s health status, in addition to being the most significant predictor of the total cost per cancer patient admitted to individual palliative care programs [29,30,31]. 

Since, in Italy, no regulatory cut-off thresholds have been established with regard to the weekly per patient visits and their relationship with the case-mix of assisted patients, our Stage 4 analysis will assess the robustness of such relationship by comparing, at the HPCS level, the observed value (weekly visits of physicians and nurses per deceased cancer patient) with its predicted value, using the mean Intensity Care Coefficient (as a proxy for the average HPCS severity), as a predictor in a statistical model (polynomial/linear regression). 

A typical measure used to identify outliers in bivariate/multivariate relationships relies on confidence intervals of individual predictions and on the Studentized Residuals (SR), where a strong outlier typically [32] falls beyond the range ±2.5.

The bottom part of Table 2 illustrates the variables in the relationship (labeled as Y − X) involved in Stage 4, as well as the statistical analyses used to identify the HPCS outliers.

In order to summarize the Best Practice Panel’s inclusion process, a HPCS registered in the OBPPC portal is admitted after passing all of the following stages:

Stage1 It satisfied the four Regulatory standards (HPCS status)
Stage2 It provided all variables of the minimum dataset
Stage3 The scores of all indicators K1–K6 are consistent (within the outer fence) with the distributions evaluated for all HPCSs that made it to Stage 3
Stage4 The score of weekly visits per deceased cancer patient is consistent with the mean Intensity Care Coefficient (SR within the range ±2.5) evaluated for the HPCSs that made it to this stage (Best Practice Panel status)

### 2.3. Process/Outcomes Measures

Finally, with regard to the HPCSs admitted within the Best Practice Panel, we examined main process measures, and particularly: the percentage of assisted cancer and pediatric patients and, for deceased cancer patients, the mean duration of care and the mean Intensity Care Coefficient; with regard to the outcome measures (referring to symptom management, quality of life and emotional concerns of family caregivers), the data allowed the evaluation of aggregated outcome measures, such as the promptness of care [11,33] and, for deceased cancer patients, the place of death [6]. 

## 3. Results

### 3.1. Registered HPCSs

Globally, in the 2014 call, 118 registered HPCSs enrolled in the study, thus 66.6% of the 177 HPCSs active across the Italian territory in 2013, as identified by Agenas.

All Italian Regions (with the exception of Molise) and the autonomous provinces (Trento and Bolzano) were represented, globally accounting for 60 provinces out of 110 and including the largest Italian cities: Milan (nine HPCSs), Rome and Turin (eight each), Florence (six), Venice and Bologna (three each) and Naples (two).

**Figure 1:**
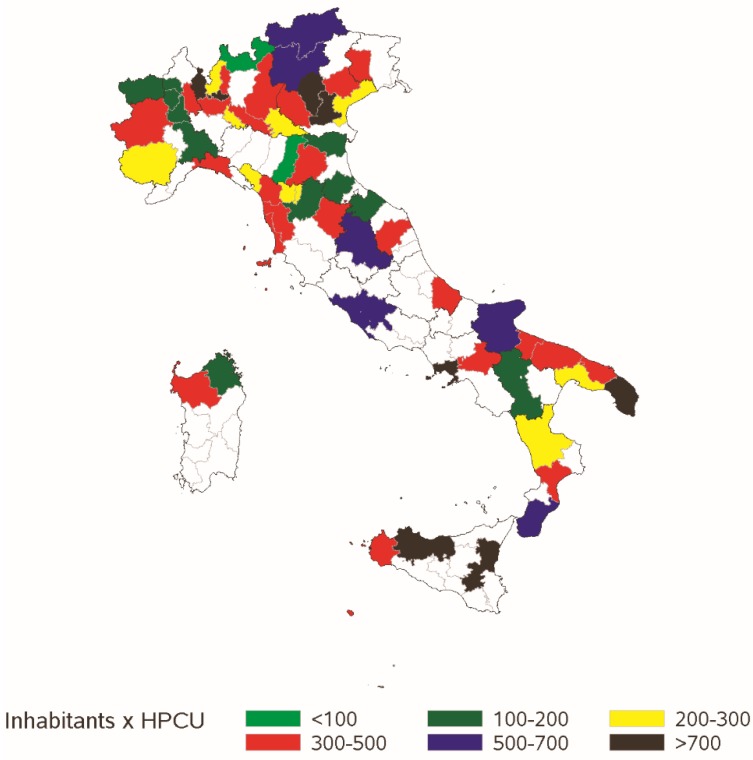
HPCSs enrolled in the study by Italian province (colored areas) and number of inhabitants (thousands) per HPCS in each province in 2013 (Regions-bold contours).

Figure 1 shows the presence of the HPCSs registered in each Italian province (colored areas) as well as the number of inhabitants per active HPCS, as a proxy for a potential basin area. 

According to 2013 official data (Italian Institute of Statistics, ISTAT, Rome, Italy), 72% of Italians live in a province covered by at least one of the 118 HPCSs (this number increases to 83% in the northwest). 

Table 1 illustrates the main domains that were investigated for the HPCSs that enrolled in the study. 

### 3.2. Best Practice Panel Admission Process

Globally, 95 out of 118 HPCSs (80.5%), met all four regulatory requirements and have a regulatory HPCS status (Stage 1): the first indicator (“The HPCS is a structure identified within the organizational plan”) had the lowest passing rate (86%).

According to the data, of almost all quality domains included in the questionnaire, the HPCSs that did not pass Stage 1 had a higher number of missing responses and significantly worst performances compared to the ones that overcame this stage, especially with regard to the provision of specialized interventions (two side *z*-test, *p* < 0.001), continuity of care (*p* < 0.001), implementation of formalized protocols to inform patients about the prognosis (*p* = 0.008) and implementation of audit programs (*p* = 0.01).

At Stage 2, 83 HPCSs (out of the 95 that made it through Stage 1, 70.3%) submitted the required minimum dataset. 

**Figure 2:**
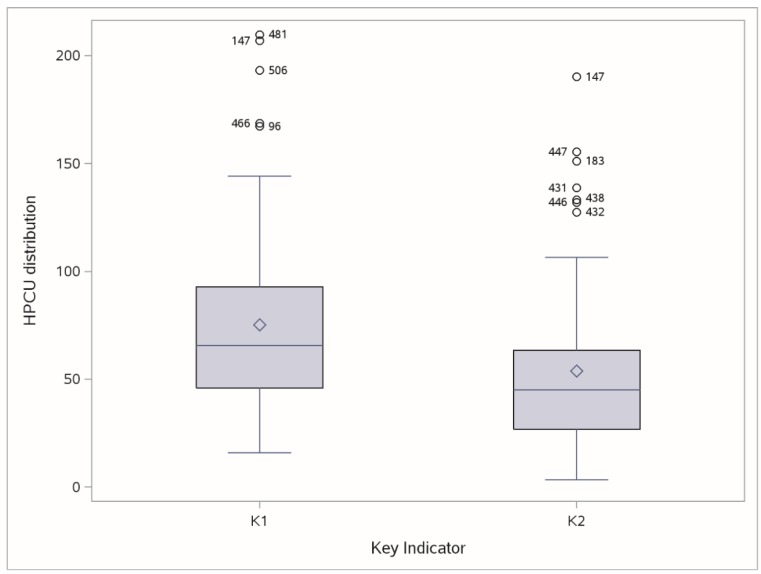
Boxplot of K1 (N = assisted cancer patients × full-time palliative care physician) and K2 (N = assisted cancer patients × full-time palliative care nurse): HPCS IDs labeled as extreme outliers (outside the outer fences).

At Stage 3, only 58 HPCSs out of the 83 that made it through Stage 2 (69.9%) showed evidence of reliable indicators (by presenting all six indicators K1–K6 within a reliable range): instead, other HPCSs showed evidence of some extreme outliers, at least for one of six indicators; for example, Figure 2 shows the HPCSs (labeled according to their ID code) considered to be outliers based on two key indicators (K1, K2).

The Stage 3 selection was confirmed by experts of the Steering Committee, since the thresholds identifying the extreme outliers for six K1–K6 indicators (K1: 148, K2: 105, K3: 1015, K4: 1020, K5: 3.5, K6: 6.2) were not considered too restrictive, suitably identifying only extreme deviations from the overall behavior. 

In Stage 4, we empirically assessed the relationship of Weekly visits (Y) based on the mean Intensity Care Coefficient (X), measured for deceased cancer patients. A linear model confirmed the expectations of a highly significant and increasing relationship (*t*-value = 4.04, *p* = 0.0002, R-square 0.241). The steering committee confirmed the validity of such empirical relationship and its robustness (with respect to outliers’ identification).

**Figure 3:**
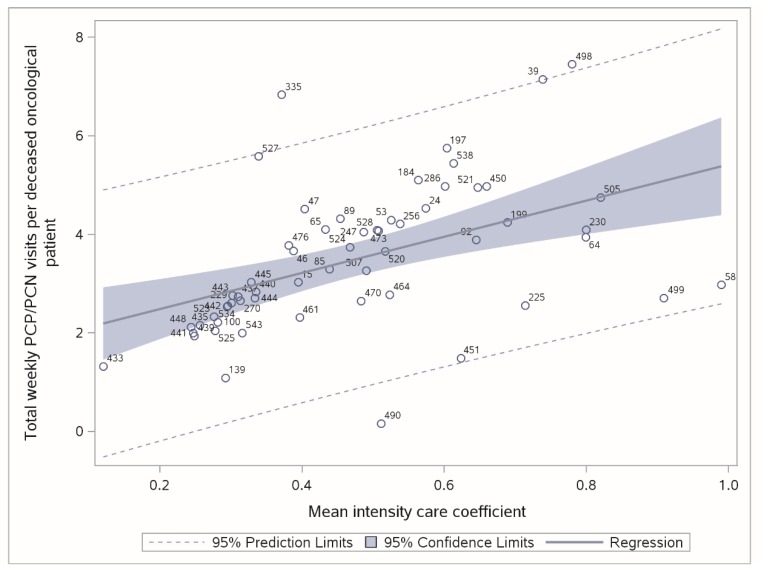
Regression plot and confidence interval of individual predictions (Stage 4). Labeled as HPCS IDs.

Figure 3 illustrates the linear regression plot (as well as the 95% confidence interval of individual predictions, as dashed lines) and two strong outliers (ID 335 and ID 490 and the related studentized residuals of 3.29 and −2.79, respectively). 

Globally, 56 HPCSs (47.5% of 118 eligible) were admitted to the Best Practice Panel. Table 3 summarizes the admission process: Stage 1 and 3 appeared as the most selective. The low mortality rate in the last stage confirms the data quality reliability for the HPCSs that made it to this stage, after the first formal data checking of Stage 3. 

### 3.3. Best Practice Panel Process and Outcomes

Table 4 shows the main process/outcome for the 56 HPCSs admitted in the Best Practice Panel.

With regard to the type of institutions, 52% belong to public structures (30% are managed by Local Health Agencies, 22% by Hospitals), whereas 48% are private institutions (almost entirely non-profit organizations, with only one private for profit hospitals). The admission rate was not found to be significantly related to type of institution (two sides *t*-test, *p*-value = 0.232). 

## 4. Discussion

This paper provided an overview of the development and activities of HPCSs in Italy. 

Despite the limitations of the study (voluntary participation) and the reliability of the data provided by the registered HPCSs, the study demonstrated a good degree of HPCS participation at a national level. 

The data provided a robust and representative survey on home palliative care facilities in Italy. Overall, in 2013, nearly 40,000 patients were assisted at home with palliative care provided by registered HPCSs. These findings are indeed consistent with those published in Institutional reports [33]. 

With regard to the selected panel of HPCSs, main empirical findings (Table 4) provided reliable data for different important domains. 

A large amount (84.4%) of deceased cancer patients had been enrolled in HPCSs within 3 days from referral (a national regulatory standard), yet a significant proportion of patients were enrolled beyond that 3-day term.

The deceased cancer patients had been visited by nurses approximately 2.2 times per week and approximately 1.3 times per week by physicians. This finding is consistent with the number of nurses employed by the at-Home Palliative Care teams.

For deceased patients (0.48), the mean intensity of care coefficient is consistent with the Italian regulatory standards and, for such subsample, the mean duration of care (61 days) corresponds to the one reported by other palliative care observers, with a value included between the one recorded by the Australian PCOC (mean 38.3 days) [34] and by the English MDS (about 100 days) [35].

Finally, with regard to the main outcome for deceased patients, a large proportion of assisted patients died at home, (respectively nearly 75% and 80% for cancer and non-cancer patients) whereas a lower percentage of deaths occurred at the hospital (around 10%). 

### Access to Quality At-Home Palliative Care Services

According to the Best Practice Panel, empirical data suggest that at-home palliative care services are still not available in the same proportions across geographic areas (more than a quarter of Italians live in a province not covered by HPCS, typically in the South and on the west coast) and according to the different diagnoses (non-cancer and pediatric patients represent a negligible percentage of assisted patients; 5% and 0.4%, respectively).

The extension of palliative care services to non-cancer and pediatric patients with complex and advanced chronic conditions, as recognized internationally [3,36,37], is still inadequate in Italy, where a cancer diagnosis remains one of the main requirements necessary for gaining access to palliative care services (wherein these patients represent about 75% of the patients under palliative treatment in Europe [38]). 

From a historical point of view, the main barriers involved in providing palliative care to non-cancer patients are related to the difficult management of the unpredictable evolution of diseases and to some difficulty in identifying a terminal stage. In fact, the patients in need of palliative care have multiple problems and symptoms, and are often referred from one service to another, therefore receiving care from a variety of professionals [39]. 

Strategic guidelines must be addressed to foster the organizational plan and to create, develop and strengthen dedicated and specialized teams by using a case management approach [40] capable of ensuring collaboration between the patients, professionals, caregivers and health-care organizations. 

Moreover, empirical data on the admission process suggests that the quality of at-home palliative care services significantly varies from one facility to another.

The small percentage of HPCSs admitted to the Best Practice Panel (47.5%), with respect to those who voluntarily joined the web portal, not only highlights how difficult it is for the Italian HPCSs to meet the requirements of the Italian NL 38/2010 in terms of the organizational criteria (80.6% of HPCSs made it through Stage 1), but also how difficult it is for them to provide of complete and reliable activity data. 

This is indicative of the HPCSs’ unwillingness to document and analyze their activities, through evaluations done according to specific processes in terms of quality, and which currently represent the strongest barrier to the benchmarking and continuous improvement of palliative care services.

In this perspective, the efforts of the management of the health services should be focused on providing structures with Information systems for the collection of data and to assess the daily activities, consistently with the standards prescribed by the regulation, in order to provide administrative information and clinical governance analyses.

In order to improve the organization of palliative care and to ensure integrated management and an adequate response to the patients’ needs, monitoring tools must be developed to evaluate and improve the activities and processes of home care through more findings and interdisciplinary research studies (especially on the processes and outcomes) of international recognition [36,37], as the same are essential to development and continuous improvement of HPCSs [41], especially in a complex sector such as palliative care, which has only recently been recognized as an official discipline in Italy.

According to a Public Health perspective [37,41], a further area of possible improvement is related to the use of early identification tools, which would allow an early detection of the patients who may need palliative care, regardless of their underlying diseases. These changes clearly require the upgrading of the HPCSs’ teams, consistent training programs and appropriate assessment tools.

## 5. Conclusions

Results exposed in the present paper demonstrate that quality Palliative care in daily practice is still a challenge for Italy. 

Notwithstanding, although much remains to be improved, in regard to both accessibility and quality/normative standards, the approach followed in the present paper suggests an extremely cost-effective method of receiving timely, relevant information from administrative data, offering regional and national stakeholders the opportunity to gain a deeper understanding of the problematic areas for home palliative care services.

## Figures and Tables

**Table 1:** Main data (N = 118 registered HPCSs, eligible for the “Best Practice Panel”).

**Stage 1: Regulatory Standards for HPCS Status**				**Label**	**%YES**
The HPCS is a structure identified within the organizational plan (simple or complex, as required by law CCNL 1998/2001, art. 27)				I1	86%
The HPCS has a team of full-time palliative care Physicians (75% of the work hours were exclusively dedicated to PC throughout the year)				I3	91%
The HPCS has a team of full-time palliative care Nurses (75% of the work hours were exclusively dedicated to PC throughout the year)				I4	92%
The HPCS adopts the Integrated Individual Care Plan (PAI) to assess the needs, to plan and implement interventions and to evaluate the outcomes of the care provided				I6	99%
**Stage 2: Minimum dataset in the Business Year 2013**	**Label**	**N**	**Total**	**Mean**	**Median**
Assisted patients	E1	118	40,955	347.0	236.5
Cancer patients	E2	117	38,384	328.7	213.0
Deceased cancer patients	Y2	117	29,008	247.9	176.0
Days with at least one home visit (deceased cancer patients)	E8_1	108	722,744	6692.7	3683.5
Duration of care, in days (deceased cancer patients)	E8_2	108	1,679,809	15553.7	8040.5
Home visits by PC physicians (deceased cancer patients)	E16_1	108	722,744	6692.0	3683.5
Home visits by PC nurses (deceased cancer patients)	E16_2	104	419,201	4030.7	2299.0
PC physicians active in the HPCS	PCP	118	2593	21.9	20.0
Yearly work hours of the PC physicians active in the HPCS	W_PCP	118	21,044	178.3	104.5
PC nurses active in the HPCS	PCN	118	2662	22.5	20.0
Yearly work hours of the PC nurses active in the HPCS	W_PCN	118	28,133	238.4	162.0
**Main Structure Data**					
Type of organization and structure of the HPCS’s organizational plan	Public 59% (36% Local Health Agencies, 23% hospitals), Private 41% (35% non-profit, 6% private hospitals)				
Type of PC provided	Specialized PC: 97% (Primary PC too: 65%)				
The HPCS is an organization that also provides palliative care in other settings (multiple answers)	Hospital and residential facilities (84%), hospices (40%), outpatient settings (58%)				
Presence of a local Palliative Care network (formalized partnership protocols in the same territorial area with): multiple answers	Local hospitals and hospices (90%), General Practitioners and Pediatricians (64%), informally with GPs for the implementation of an Individual Care Plan (80%)				
The HPCS provides pediatric care	76%				
Formalized support of Non-Profit Organizations	69%				
Home Beds Equivalent (total days of care in the year/number of days in the year)	1–10: 12%, 11–20: 17.8%, 21–30: 7.6%, 31–40: 14.4%, 41–60: 13.6%, 61–100: 15.3%, ≥100: 19.4% (≥200: 7.6%)				
**Continuity of the Care**					
Is the continuity of care guaranteed on weekends and nights?	Not by		Only phone by	Guaranteed by	
	Nurse—Doctor		Nurse—Doctor	Nurse—Doctor	
Night:	69.5%—33.1%		6.8%—20.3%	23.7%—46.6%	
Saturday noon:	8.5%—20.3%		5.9%—14.4%	85.6%—65.2%	
Sunday morning:	8.5%—20.3%		1.7%—13.6%	89.8%—66.1%	
Sunday noon:	14.4%—15.3%		5.9%—13.6%	79.6%—71.2%	
**Main Process Data**					
**Meetings, Training and staff supervision of the HPCSs:** 76% have weekly meetings to discuss the Individual Care Plan, 12% depend on prognosis, 12% once a month. 93% organize specific training to the medical and nursing staff, 83% guarantee periodic psychological support to the team.					
**Clinical care procedures of the HPCSs:** 93% adopt procedures to **evaluate patients’ needs** and to modify the Individual Care Plan. 57% rely on a structured **interview with families** before assisting patients, 40% are not structured and 3% have no interviews. 13% rely on a structured **interview directly with the patient**, 69% are not structured and 19% have no interviews. HPCSs **use standardized scales** to evaluate pain (99%, 39% pediatric care), physical symptoms (81%), quality of life (64%, 3% pediatric care) and psychological symptoms (30% patient, 16% family). Patients and families’ **non-clinical needs**: HPCSs guarantee psychological support (87%), spiritual assistance (38%), social/economic-related support (38%). At the near death **stage**, HPCSs adopt standardized tools to evaluate patients’ needs (91%), to communicate with the families (88%), to evaluate the symptoms (92%), to provide support (84%). 79% collaborate with an ethical committee, 32% adopts structured procedures for ethical conflicts. 8% does not supply drugs (opioids) at home.					
**Informative systems (ITC) and Quality initiatives:** 96% use ICT to record the activity data, 69% use ICT to record and monitor the activity data, 86% use patient satisfaction questionnaires, 80% use risk management procedures, 79% use audit/feedback reports to continuously improve.					

**Table 2:** Key indicators (K1–K6) and relationship (R) in Stages 3 and 4, respectively.

**Stage 3: Key Indicators**	**Label**	**Formula**	**Outlier Check**
Assisted cancer patients × FTPCP	K1	E2/FTPCP ^a^	Boxplot, outer fence
Assisted cancer patients × FTPCN	K2	E2/FTPCN ^b^	Boxplot, outer fence
FTPCP home visits × 100 deceased cancer patients	K3	[(E16_1/FTPCP)/Y2 ^c^] × 100	Boxplot, outer fence
FTPCN home visits × 100 deceased cancer patients	K4	[(E16_2/FTPCN)/Y2 ^c^] × 100	Boxplot, outer fence
Weekly PC Physician visits × deceased cancer patient	K5	(E16_1/Y2)/TW ^d^	Boxplot, outer fence
Weekly PC Nurse visits × deceased cancer patient	K6	(E16_2/Y2)/TW ^d^	Boxplot, outer fence
**Stage 4: Relationship to Monitor**	**Label**	**Statistical Analysis**	**Outlier Check**
Weekly visits x deceased cancer patient (Y = K5 + K6) and mean intensity care coefficient (X = E8_1/ E8_2) ^c^	Y − X	Y vs. predicted value (Polynomial regression)	Prediction bands, studentized residuals

^a^ FTPCP = Number of full-time (38 h a week) palliative care physicians within the team, in a week; ^b^ FTPCN = Number of full-time (36 h a week) palliative care nurses within the team, in a week; ^c^ For Y2, E8_1 and E8_2, see Table 1; ^d^ TW = (E8_2/Y2)/**7**, mean length of care (weeks) × deceased cancer patient

**Table 3:** Summary of the admission Process of the 118 registered HPCSs.

Admission Process		HPCS that Passed	
Stage	Label	N	% of 118
Stage 1	Regulatory status of the HPCSs	95	80.6%
Stage 2	Provision of the Minimum dataset	83	70.3%
Stage 3	Reliable indicators (K1–K6 no outliers)	58	49.2%
Stage 4	Reliable empirical ratio (Y − X)	56	47.5%

**Table 4:** Main activity indicators and outcome (N = 56 HPCSs of the Best Practice Panel).

Activity Data and Main Indicators	N	Min	Mean	Median	Max
Home Beds Equivalent Class	56	11–20	na	31–40	≥200
Assisted patients (N = 19,866)	56	27.0	354.6	275.5	2141.0
% pediatric patients per patients assisted	53	0.0	0.4	0.0	3.7
Cancer patients (N = 18,858)	56	27.0	336.8	263.0	2054.0
% cancer patients per patients assisted	56	38.6	94.9	100.0	100.0
Deceased cancer patients (N = 15,512)	56	14.0	259.1	210.0	1591.0
% deceased cancer patients per cancer patients	56	25.2	77.4	82.9	100.0
Non-cancer patients (N = 1008)	56	0.0	17.9	0.0	208.0
Deceased non-cancer patients (N = 623)	54	0.0	11.5	0.0	184.0
FTPCPs × team	56	0.8	3.7	3.0	12.0
FTPCNs × team	56	1.7	5.6	4.6	24.8
Patients assisted × FTPCP	56	17.1	113.8	81.0	433.0
Patients assisted × FTPCN	56	11.4	73.4	57.6	190.4
Cancer patients × FTPCP	56	17.1	103.0	77.2	337.3
Cancer patients × FTPCN	56	7.6	69.5	55.0	123.5
Deceased cancer patients × FTPCP	56	8.9	83.3	58.5	300.3
Deceased cancer patients × FTPCN	56	5.6	53.8	41.0	115.7
Duration of care in days (deceased cancer patients)	56	17.0	60.7	49.8	126.3
Mean Intensity care coefficient (deceased cancer patients)	56	0.12	0.48	0.46	0.99
Home visits × FTPCP per patient assisted	55	0.0	3.4	2.7	8.9
Home visits × FTPCN per patient assisted	55	0.0	4.1	3.3	13.4
Home visits × FTPCP per deceased cancer patient	56	0.3	3.7	2.9	12.1
Home visits × FTPCN per deceased cancer patient	56	0.8	4.0	3.5	10.1
Weekly visits at home of FTPCPs × deceased cancer patient	56	0.2	1.3	1.3	3.0
Weekly visits at home of FTPCNs × deceased cancer patient	56	0.5	2.2	2.1	5.8
Promptness of take in charge (deceased cancer patients)					
≤1 day from the report	51	0.0	24.2	1.7	100.0
≤2 days from the report	51	0.0	17.3	6.9	100.0
≤3 days from the report	51	0.0	43.0	17.3	100.0
>3 days from the report	51	0.0	15.6	1.1	100.0
Place of death (deceased cancer patients)					
Home	55	33.8	74.4	78.8	100.0
Hospice	55	0.0	14.2	11.1	51.5
Hospital	55	0.0	10.7	9.1	37.9
Nursing homes	55	0.0	0.7	0.0	10.1
Place of death (deceased non-cancer patients)					
Home	22	25.0	79.6	87.2	100.0
Hospice	22	0.0	7.6	0.0	40.0
Hospital	22	0.0	10.1	2.9	75.0
Nursing homes	22	0.0	2.7	0.0	33.3

^a^ Days with at least one home visit/duration of care in days, average per HPCS.
